# *In vitro* and *in vivo* pharmacokinetic characterization of LMT-28 as a novel small molecular interleukin-6 inhibitor

**DOI:** 10.5713/ajas.19.0463

**Published:** 2019-08-03

**Authors:** Sung-Hoon Ahn, Tae-Hwe Heo, Hyun-Sik Jun, Yongseok Choi

**Affiliations:** 1 College of Pharmacy, Kangwon National University, Chuncheon 24341, Korea; 2 Laboratory of Pharmacoimmunology, Integrated Research Institute of Pharmaceutical Sciences, and BK21 PLUS Team for Creative Leader Program for Pharmacomics-based Future Pharmacy, College of Pharmacy, The Catholic University of Korea, Bucheon 14662, Korea; 3 ILAb Inc., NP513, College of Pharmacy, The Catholic University of Korea, Bucheon 14662, Korea; 4 Department of Biotechnology and Bioinformatics, College of Science and Technology, Korea University, Sejong 30019, Korea; 5 School of Life Sciences and Biotechnology, Korea University, Seoul 02841, Korea

**Keywords:** Interleukin-6, gp130, Pharmacokinetics, Metabolic stability, Inflammation

## Abstract

**Objective:**

Interleukin-6 (IL-6) is a T cell-derived B cell stimulating factor which plays an important role in inflammatory diseases. In this study, the pharmacokinetic properties of LMT-28 including physicochemical property, *in vitro* liver microsomal stability and an *in vivo* pharmacokinetic study using BALB/c mice were characterized.

**Methods:**

LMT-28 has been synthesized and is being developed as a novel therapeutic IL-6 inhibitor. The physicochemical properties and *in vitro* pharmacokinetic profiles such as liver microsomal stability and Madin-Darby canine kidney (MDCK) cell permeability assay were examined. For *in vivo* pharmacokinetic studies, pharmacokinetic parameters using BALB/c mice were calculated.

**Results:**

The logarithm of the partition coefficient value (LogP; 3.65) and the apparent permeability coefficient values (P_app_; 9.7×10^−6^ cm/s) showed that LMT-28 possesses a moderate-high cell permeability property across MDCK cell monolayers. The plasma protein binding rate of LMT-28 was 92.4% and mostly bound to serum albumin. The metabolic half-life (t_1/2_) values of LMT-28 were 15.3 min for rat and 21.9 min for human at the concentration 1 μM. The area under the plasma drug concentration-time curve and C_max_ after oral administration (5 mg/kg) of LMT-28 were 302±209 h·ng/mL and 137±100 ng/mL, respectively.

**Conclusion:**

These data suggest that LMT-28 may have good physicochemical and pharmacokinetic properties and may be a novel oral drug candidate as the first synthetic IL-6 inhibitor to ameliorate mammalian inflammation.

## INTRODUCTION

The discovery and development of a new drug can save millions of lives and enhance the quality of numerous lives. Although the pharmaceutical and biotechnical researches and developmental efforts may lead to successful approval, there are also many hurdles in drug discovery and development [1]. Most of all, in order to obtain regulatory approval, we have to discover a drug candidate which shows proper efficacy, safety, and drug-like properties [2,3]. A good strategy for achieving successful clinical trials is to determine absorption, distribution, metabolism, excretion, and pharmacokinetics of a drug including physicochemical properties such as LogP and solubility [4,5].

Activation of the interleukin-6 (IL-6) and signal transducer and activator of transcription factor-3 (STAT3) signaling pathway have been studied and IL-6, a phosphorylated glycoprotein, is a major causative factor which plays an important role in inflammatory disease [6,7]. IL-6 plays an important role in anti-inflammatory and host defense and overexpression of IL-6 due to pathogens, stresses, and aging may lead to the pathogenesis of inflammatory processes, autoimmune response, and cancer [8–10]. The therapeutic antibody (Ab) agents which inhibit IL-6 have been developed and showed effective and potential alternatives for inflammatory diseases refractory to conventional drugs. Recently, clinical studies have either been done or are ongoing on therapeutic antibodies such as sirukumab (CNTO136), siltuximab (CNTO328), olokizumab (CP6038), elsilimomab (BE-8), clazakizumab (BMS945429), PF-423691, and MED I5117, and by anti-IL-6R Abs including sarilumab (REGN88) and tocilizumab (Actemra) [11]. Even though these mAb biologics targeting IL-6 have been showing success in commercial view, they have several issues such as high cost, invasive administration, and high rate of immunogenicity. Therefore, the discovery and development of new oral absorptive small molecular inhibitors are required, particularly with the attributes of low toxicity and low antigenicity.

**Figure 1:**
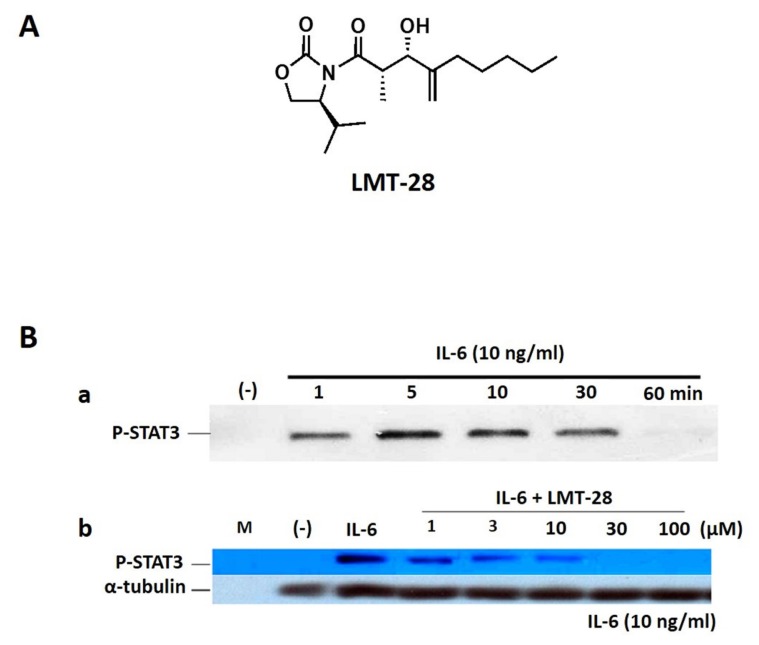
(A) Chemical structure of LMT-28. (B) Inhibition of IL-6-induced STAT3 activation by LMT-28. (a) HepG2 cells were treated with IL-6 alone (10 ng/mL) for 1, 5, 10, 30, or 60 min, and phosphorylated-STAT3 (p-STAT3) was detected by Western blotting (WB). (b) HepG2 cells were treated with LMT-28 (1, 3, 10, 30, or 100 μM) for 1 h and then were stimulated for 10 min with IL-6 (10 ng/mL). Cells were collected, lysed, and analyzed for p-STAT3 by WB. α-Tubulin was detected as a loading control. STAT3, signal transducer and activator of transcription factor-3.

To discover a novel synthetic small molecule as a new drug candidate for IL-6 blocking, LMT-28 (Figure 1A) has been screened, identified from chemical library and investigated for its activity and mechanism of action [12–15]. LMT-28 has an inhibitory effect on the activation of STAT3 induced by IL-6. LMT-28 also has a down-regulatory effect for IL-6-stimulated phosphorylation of STAT3, gp130, and JAK2 protein. In addition, LMT-28 directly interacts with gp130 and specific reduction of IL-6/IL-6Rα complex binding to gp130. Taken together, it is reasonable that LMT-28 can be a first synthetic IL-6 inhibitor with IL-6 inhibitory activity and non-cytotoxicity.

The aim of this study was therefore to evaluate pharmaco kinetic profiles of LMT-28 as a novel IL-6 inhibitor, because there are no reports about characterizing it as a synthetic small molecule that inhibits IL-6 and ameliorates inflammation in mice by functioning through direct interaction with gp130. Furthermore, both *in vitro* and *in vivo* pharmacokinetic profiling including physicochemical properties were characterized to support future development and clinical trials.

## MATERIALS AND METHODS

### Chemicals

LMT-28 (Figure 1A, M.W. 311.42), an oxazolidinone derivative, was synthesized with a purity >99.0%. Liver microsome and nicotinamide adenine dinucleotide phosphate hydrogen (NADPH) regeneration solution for metabolic stability were purchased from Corning Gentest (Tewksbury, MA, USA). Carbamazepine as an internal standard along with other reagents were purchased from Sigma-Aldrich (St. Louis, MO, USA). Water, acetonitrile and other solvents high-performance liquid chromatography (HPLC) grade or the highest quality were purchased from J.T. Baker (Phillipsburg, NJ, USA).

### Animals

Male BALB/c mice (CD-1) were purchased from Orient-Bio Company (Seongnam, Korea). Animals were kept in an air conditioned room at a temperature of 20°C to 25°C, humidity (50%±3%), and 12 h day/night cycles with specific free condition. Food and water were supplied *ad libitum*. Animals were fasted for 12 h before experiments while still having access to water. All animal procedures involving animal care were approved by the Institutional Animal Care and Use committee at Kangwon National University (approval no. KW-151127-2).

### Western blot analysis for inhibitory effect of interleukin-6-induced signaling

HepG2 cells were seeded in MEM media (Welgene, Gyeongsan, Korea) with 10% (v/v) feta bovine serum, streptomycin (100 U/mL), and penicillin (100 U/mL; Life Technologies, Carlsbad, CA, USA). After seeding, cells were starved overnight, treated with LMT-28 for 1 h, and stimulated with IL-6 (10 ng/mL; R&D Systems, Minneapolis, MN, USA) for 10 min. Cells were treated with cell lysis buffer (Promega, Madison, WI, USA) for 30 min on ice. Cell lysates were separated on an 8% sodium dodecyl sulfate-polyacrylamide gel electrophoresis gel, then transferred to nitrocellulose membranes (Schleicher & Schuell, Dassel, Germany). The membranes were blocked overnight in Tris-buffered solution (50 mM Tris-HCl [pH 7.4], 150 mM NaCl, 0.1% Tween 20) containing 5% nonfat dry milk at 4°C and then incubated for 1 h with the appropriate primary Abs against p-STAT3 (MilliporeSigma, Burlington, MA, USA) or α-tubulin in Tris-buffered solution (TBST; 50 mM Tris-HCl [pH 7.4], 150 mM NaCl, 0.1% Tween 20) containing 5% nonfat dry milk at room temperature. The membranes were washed (10 min, five times) and incubated for 1 h with horseradish peroxidase-conjugated secondary Abs diluted to 1:6,000. After 10 washes, the membranes were incubated with enhanced chemiluminescence reagents (MilliporeSigma, USA) and chemiluminescent signals were visualized using X-ray film.

### Physicochemical properties of LMT-28

The shake-flask method was applied to measure the equilibrium solubility of LMT-28 [16,17]. Excess amount of LMT-28 was added into the water and the suspension was shaken at 25°C during 24 h in a flask for equilibrium solubility. The solution, then, was filtered and the concentration of LMT-28 in the filtered solution was quantified using ultra HPLC (ThermoFisher Scientific, Waltham, MA, USA) at wavelength 254 nm. The lipophilicity of LMT-28 was measured by using GLpKa (Pion, Cambridge, MA, USA) based on dip-probe absorption spectroscopy (D-PAS) method. The resultant values were calculated by LogP software (Pion, USA) and the computational predictions for number of hydrogen bond donors, hydrogen bond acceptors, rotatable bonds and predicted polar surface area (PSA) and LogS were made using Percepta software (ACD/Labs; Toronto, Canada).

### Metabolic stability of LMT-28

Metabolic stability using rat and human liver microsomes (Corning Gentest, USA) of LMT-28 was performed according to previous reported protocol [18]. In brief, NADPH regeneration solution (Corning Gentest, USA) was added to initiate the metabolic reaction after mixing LMT-28 (final concentrations; 1 μM) with human or rat liver microsomes (0.5 mg protein/mL) in 100 mM potassium phosphate buffer (pH 7.4). After incubation at 37°C at 0, 10, 30, and 60 min, the reaction was terminated by adding three times of ice-cold acetonitrile containing internal standard (carbamazepine 10 ng/mL). After mixing by vortex and centrifugation at 10,000 g for 5 min at 4°C, the supernatants were transferred to fresh LC vials. Samples (5 μL) were analyzed by directly injection into a liquid chromatography-tandem mass spectrometry (LC-MS/MS) system for the quantification of LMT-28.

The metabolic half-life and *in vitro* intrinsic clearance of LMT-28 were calculated by next equations [19].

$$\begin{array}{l} \text{Half-life }({\text{t}}_{1/2})\;(\text{h})=-0.693/\text{K}\\ {\text{CL}}_{\text{int},in\;vitro}(\mu \text{L}/\text{min}/\text{mg protein})\\ =(0.693/{\text{t}}_{1/2,\text{microsomal}})\times (\mu \text{L incubation}/\text{mg microsomal protein})\\ {\text{CL}}_{\text{int},in\;vivo}(\text{mL}/\text{min}/\text{kg})\\ =(0.693/{\text{t}}_{1/2,\text{microsomal}})\times (\text{mL incubation}/\text{mg microsomal protein})\\ \times (\text{mg microsomal protein}/\text{g liver})\times (\text{g liver}/\text{kg body weight}) \end{array}$$

where K is the elimination rate constant of LMT-28 in the microsomal metabolic reaction, half-life (t_1/2_) is microsomal metabolic half-life, CL_int, *in vitro*_ is *in vitro* intrinsic clearance, and CL_int, *in vivo*_ is *in vivo* intrinsic clearance. The liver weight relative to the body weight in the rat and human are 40 and 21 g/kg, respectively [20].

### *In vitro* pharmacokinetic study of LMT-28

In order to determine cell permeability of LMT-28, Madin-Darby canine kidney (MDCK) cells were cultured and seeded at a density of 6×10^4^cells/cm^2^ in 12-well Transwell plates. After checking cell monolayer integrity, the transepithelial electrical resistance (TEER) values (>500 ohms) were measured using an EVOM epithelial voltohmmeter (World Precision Instruments, Sarasota, FL, USA). After pre-incubation for 30 min at 37°C, LMT-28 (final concentrations of 2, 10, and 50 μM) was added to the donor wells in transport assay system. Sample aliquots (100 μL) were collected every 30 min for 120 min, and the same volume of fresh buffer added. The aliquots were stored at −20°C until quantitative analysis of LMT-28 concentrations by LC–MS/MS system. The apparent permeability coefficient (P_app_, cm/s) was calculated as following equation:

$${\text{P}}_{\text{app}}=(\text{dQ}/\text{dt})/(\text{A}\times {\text{C}}_{0})$$

where dQ/dt is the permeation rate across cell monolayer, A is the surface area of the monolayer (0.33 cm^2^), and C_0_ is the initial donor concentration.

### *In vivo* pharmacokinetic study of LMT-28 in mice

To evaluate *in vivo* pharmacokinetics in mice, LMT-28 was dissolved in phosphate-buffered saline (pH 7.4) containing 5% (w/v) carboxymethyl cellulose (MilliporeSigma, USA) and administered orally by using oral needle and intravenously in the mouse tail vein at the dose of 5 mg/kg to male BALB/c mice (22 to 25 g) after overnight fasting. Blood samples (50 μL) were collected at 0.5, 1, 2, 4, 6, and 8 h after oral administration of LMT-28. After taking the blood samples from mice, the samples were centrifuged to obtain plasma samples at 4°C and 10,000 g and obtained plasma samples were stored at −20°C. These plasma samples (25 μL) were used for the quantification of LMT-28 concentration in mice.

### Quantitative analysis of LMT-28 in biological fluids using liquid chromatography-tandem mass spectrometry

The concentrations of LMT-28 in plasma or microsomal reaction solution were determined by LC-MS/MS. For the sample preparation before LC-MS/MS analysis, three times (75 μL) of acetonitrile containing internal standard (carbamazepine 10 ng/mL) were added to 25 μL aliquots of the plasma samples. The samples were vortex-mixed and centrifuged at 10,000 g for 5 min at 4°C for the protein precipitation. The supernatant (90 μL) was transferred to LC vials and 5 μL was injected onto a LC-MS/MS system.

Mass spectrometric detection of LMT-28 was performed on an API 4000 Qtrap mass spectrometer (AB Sciex, Foster City, CA, USA) equipped with a positive electrospray ionization source. The chromatographic separation was performed on Hypersil Gold C18 column (50 mm×2.1 mm i.d., 3 μm particle size; ThermoFisher Scientific, USA) with a flow rate of 0.3 mL/min. The positive multiple reaction monitoring (MRM) mode were based on the most abundant product ions at m/z 312 → 165 for LMT-28, and m/z 237 → 194 for internal standard. Other parameters were the nebulizing (GS1), 50 psi; heating (GS2), 50 psi; collision energy, 23 eV for LMT-28 and 15 eV for internal standard, respectively. Analyst software version 1.4.2 (AB Sciex, USA) was used for the analysis of LMT-28 concentrations.

### Pharmacokinetic and statistical analysis

The plasma concentrations versus time profiles of LMT-28 in mice were analyzed by a non-compartmental model using WinNonlin software ver 5.3 (Pharsight, Mountain View, CA, USA). The pharmacokinetic parameters such as area under the plasma concentration-time curve (AUC), peak plasma concentration (C_max_) and the time to reach a peak concentration (T_max_), terminal elimination half-life (t_1/2_), mean residence time (MRT) after oral administration were obtained from each mouse’s plasma concentration-time plots for LMT-28. All data were expressed as the mean±standard deviation. A value of p<0.05 by student t-test or analysis of variance analysis was considered to be statistically significant.

## RESULTS

### Inhibition of interleukin-6-induced signaling by LMT-28

As shown in Figure 1, the inhibitory effect of LMT-28 on IL-6-induced signaling was investigated. First, to obtain the ideal time condition for IL-6 treatment, HepG2 cells were treated with IL-6 at a concentration of 10 ng/mL for 1, 5, 10, 30, or 60 min and p-STAT3 was detected by Western blotting (Figure 1B). HepG2 cells which were pre-treated by LMT-28 (1, 3, 10, 30, and 100 μM) for 1 h were stimulated with IL-6 at a concentration of 10 ng/mL for 10 min to examine the inhibitory effect of LMT-28. As a result, pretreatment with LMT-28 was shown to inhibit the IL-6-induced p-STAT3 expression in a dose-dependent manner (Figure 1B).

### Characterization of physicochemical properties of LMT-28

The typical physicochemical properties of LMT-28 including LogP and solubility were measured by using GLpKa (Pion, USA) based on D-PAS method and by 24 h shake-flask method based on equilibrium solubility, respectively [21]. The LogP value of LMT-28 was 3.65 and the solubility of LMT-28 in water was 0.39 mg/mL. Molecular weight of LMT-28 is 311.42, indicating that LMT-28 is very small molecule, compared to normal small molecular new drug candidates. LMT-28 is characterized by possessing one of hydrogen bond donor and five of hydrogen bond acceptors. Estimated PSA is 66.84 and number of rotatable bonds is eight. Predicted LogS is −2.96. These estimated structural physicochemical properties were predicted by Percepta software (ACD/Labs, Canada) and good values for new drug candidate by rule of five [22,23].

### Metabolic stability of LMT-28

**Figure 2:**
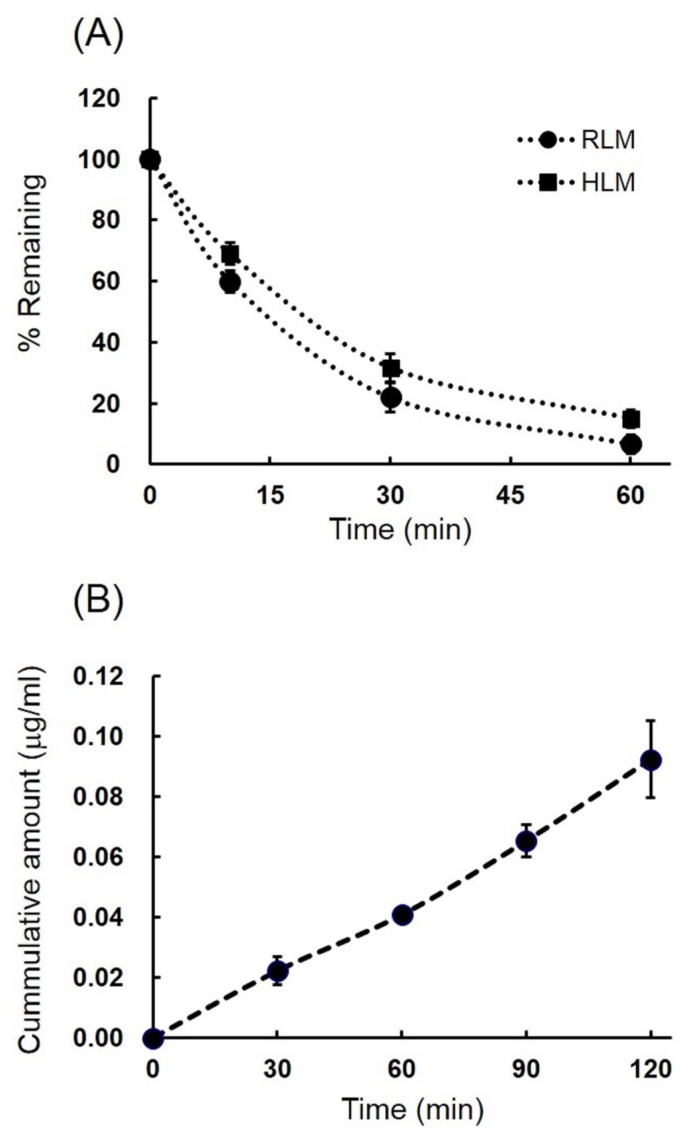
Microsomal metabolic stability of LMT-28 in rat and human (A) and MDCK cell permeability of LMT-28 (B). The remaining amount of LMT-28 (1 μM, n = 3) at 10, 30, 60 min after the microsomal incubation at 37°C were plotted in rat (circle) and human (square) microsomes. Accumulative amount of LMT-28 across MDCK cell monolayers were plotted at 30, 60, 90, 120 min after incubation in 12-well Transwell plate for transport study. Each point represents mean±standard deviation. MDCK, Madin-Darby canine kidney.

Metabolic stability in rat and human liver microsomes were measured up to 60 min as shown in Figure 2A, and metabolic stability values such as liver microsomal half-life, *in vitro* intrinsic clearance, and *in vivo* intrinsic clearance were summarized in Table 1. Liver microsomal half-life (t_1/2_) values of LMT-28 using rat and human liver microsomes were 15.3± 1.4 min and 21.9±2.8 min at the concentration 1 μM for rat and human, respectively. The calculated CL_int,_*_in vitro_* values were 91±8.4 and 64±8.1 (μL/min/mg protein) for rat and human, respectively. The CL_int,_*_in vivo_* values were also calculated by the equation in method section and those were 164±15 and 60± 7.7 (mL/min/kg body weight) for rat and human, respectively. These results suggest that LMT-28 undergoes metabolism in rat and human pooled liver microsomes at 10 μM and metabolic stability of LMT-28 in human may be higher than rat because of species difference.

### *In vitro* pharmacokinetic study of LMT-28

Important *in vitro* pharmacokinetic factors which affect drug absorption and distribution such as permeability and plasma protein binding of LMT-28 are shown in Table 1. Accumulative amount of LMT-28 across MDCK cell monolayers were plotted at 30, 60, 90, 120 min after incubation in 12-well Transwell plate for transport study and shown in Figure 2B. The P_app_ of LMT-28 at 10 μM was 9.7±1.8×10^−6^ cm/s by cell permeability using MDCK cell monolayers. The plasma protein binding rate of LMT-28 at 2 μM was 92.4%±2.1% and mostly bound to serum albumin. These results suggest that LMT-28 may be absorbed and distributed easily with affordable permeability coefficient across cell membrane and acceptable plasma protein binding ratio in systemic blood circulation.

### *In vivo* pharmacokinetics of LMT-28 in mice

**Figure 3:**
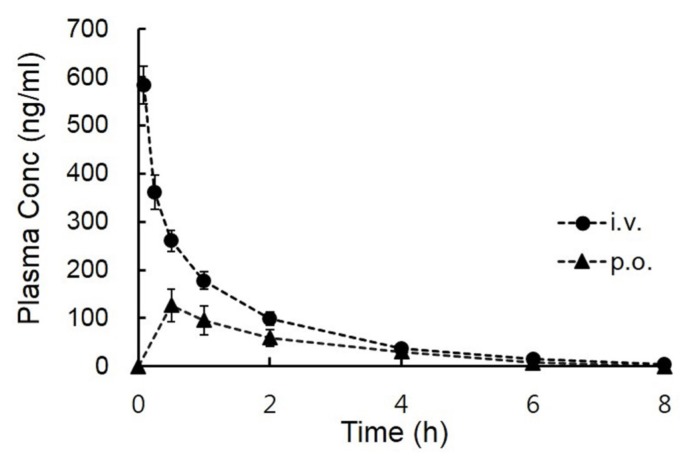
Plasma concentration-time profiles of LMT-28 after intravenous (circle) and oral (triangle) administration at doses of 5 mg/kg in male BALB/c mice (mean±standard deviation, n = 4).

After intravenous and oral administration at a dose of 5 mg/kg to male BALB/c mice (22 to 25 g), the mean plasma concentration-time curves of the LMT-28 are shown in Figure 3. Summarized pharmacokinetic parameters such as T_max_, C_max_, CL, V_ss_, AUC, MRT, and F are listed in Table 2. The pharmacokinetic values of CL, V_ss_, and AUC after intravenous administration of LMT-28 (5 mg/kg) were 8.66±4.51 L/h/kg, 12.9±4.66 L/kg, and 677±264 h·ng/mL, respectively. The pharmacokinetic values of AUC and C_max_ after oral administration of LMT-28 (5 mg/kg) were 302±209 h·ng/mL and 137±100 ng/mL, respectively. These results indicate LMT-28 was orally exposed in mice.

### Liquid chromatography-tandem mass spectrometry analysis of LMT-28 in biological fluids

The concentrations of LMT-28 in plasma or microsomal reaction solution were determined by LC-MS/MS. The LC-MS/MS analysis were performed by MRM mode using precursor ion and product ion mass spectra based on the chemical structure of analyte. LMT-28 and IS were analyzed for the abundant precursor ions [M+H]^+^ at m/z 312 and 237 and fragmented into produce product ions at m/z m/z 312 → 165 for LMT-28 and m/z 237 → 194 for carbamazepine, respectively. The chromatogram of LMT-28 and IS were eluted at 1.2 and 1.4 min with apparently sharp and symmetric peaks. A calibration curve for LMT-28 was generated by a linear least squares regression analysis by peak area ratio of LMT-28/IS chromatogram and the correlation coefficient (R^2^) ranged from 5 to 500 ng/mL to was greater than 0.99 with good linearity. LMT-28 was stable under various conditions of sample handling and processing.

## DISCUSSION

LMT-28, a new drug candidate, with the mechanism of action as a novel therapeutic IL-6 inhibitor is under development. IL-6 inhibition by LMT-28 has been demonstrated to be effective *in vitro* and *in vivo* by previous reports [15] and confirmed once again by Western blotting (Figure 1). To develop LMT-28 as a candidate for a new drug, pharmacokinetic profiling is required for preclinical and clinical studies. Thus, *in vitro* and *in vivo* pharmacokinetic properties of LMT-28 were investigated and the pharmacokinetic properties of LMT-28 were confirmed in this study.

The physicochemical properties of LMT-28 were drug-like. The equilibrium solubility of LMT-28 was 0.39±0.12 mg/mL. This was slightly soluble in water system, however, it was a reasonable solubility for a new drug candidate. More importantly, new drug candidates may require a balance between hydrophilicity and lipophilicity [24]. Log P value of LMT-28 was 3.65±0.22 by GLpKa analytical system (Pion, USA). This Log P value indicated the lipophilicity of LMT-28 may be involved in high permeability across monolayer of MDCK cells.

In liver microsomal stability, LMT-28 was eliminated at a moderate rate by both human and rat cytochrome P450 metabolic enzymes. The half-life value of rat liver microsomal stability was 15.3 min and that of human was 21.9 min. In addition, there were interspecific differences between human and rat liver microsomal stability and metabolic stability of human liver microsome was slightly higher than that of rat. These data suggested the bioavailability in humans can be increased compared to that in rats. In addition, the differences between human and rat might be caused by the genomic differences of cytochrome P450 genes between human and rodent [25,26].

In this study, MDCK cells were used to examine cell per meability, drug absorption, and tissue disposition. Even though Caco-2 cell is one of the gold standard methods to evaluate drug properties, MDCK cells are also widely used for estimating both drug absorption and disposition to tissues [3,23]. In addition, compared to Caco-2 cells, MDCK cells have some advantages such as fast growing and no leakage of cell monolayer with tight junction expression and high TEER values (>700 ohms), which makes MDCK a good choice for estimating drug absorption and tissue disposition [27,28]. However, further evaluation of drug absorption may require Caco-2 cell transport studies.

LMT-28 had very good permeability properties in the MDCK cell permeability assay. The P_app_ was 9.7±1.8 (×10^−6^ cm/s) at 10 μM. Furthermore, in the plasma protein binding assay, LMT-28 showed moderate plasma protein binding properties (92.4%). These results suggested that most of LMT-28 bound to plasma protein for transport in blood circulation. Taken together, it is reasonable to suggest LMT-28 is a good drug candidate for novel therapeutic IL-6 inhibition.

*In vivo* pharmacokinetic studies using BALB/c mice, the area under the plasma concentration-time curve values of LMT-28 during 8 h (AUC_8h_) after oral administration (5 mg/kg) was 292±202 h·ng/mL and AUC_∞_ after integration into infinity was 302±209 h·ng/mL. The sampling time (8 h) was over 7 times of terminal half-life (t_1/2_; 1.13 h) and these AUC values for 8 hours (AUC_8h_) were able to cover approximately 96.7% of the total AUC (AUC_∞_). Thus, in the case of oral administration, the sampling time and the AUC value for 8 hours were sufficient to assess *in vivo* pharmacokinetics of LMT-28. The peak plasma concentration (C_max_) was 137±100 ng/mL and the time to reach C_max_ (T_max_) was 0.80±0.67 h. After oral administration of LMT-28 to BALB/c mice, C_max_ and T_max_ showed good drug-like properties. Compared with the AUC value after intravenous administration, the bioavailability (F) of LMT-28 was calculated to be approximately 38.2%, and this may be regarded as a reasonable value considering that LMT-28 has been developed as a novel oral drug candidate for the first synthetic IL-6 inhibitor.

## CONCLUSION

In summary, LMT-28 showed a moderate liver microsomal stability in both rat and human, and the bioavailability in human may be increased due to their interspecific differences between rat and human liver microsomal stabilities. In addition, the high cellular permeability of LMT-28 across monolayers of MDCK cells may be associated with sufficient exposure to systemic blood circulation following oral administration of LMT-28 in the *in vivo* system of mice. These results suggest that LMT-28 is a good oral drug candidate for the first synthetic IL-6 inhibitor.

## Figures and Tables

**Table 1:** Physicochemical properties and *in vitro* pharmacokinetic profiles of LMT-28

Parameters	Values
LogP	3.65±0.22
Solubility in water (mg/mL)	0.39±0.12
Liver microsomal stability	
Half-life (t_1/2_, rat) (min)	15.3±1.4
Half-life (t_1/2_, human) (min)	21.9±2.8
*In vitro* intrinsic clearance	
CL_int, *in vitro*_ (Rat, μL/min/mg protein)	91±8.4
CL_int, *in vitro*_ (Human, μL/min/mg protein)	64±8.1
*In vivo* intrinsic clearance	
CL_int, *in vivo*_ (Rat, mL/min/kg)	164±15
CL_int, *in vivo*_ (Human, mL/min/kg)	60±7.7
MDCK cell permeability (P_app_, ×10^−6^ cm/s)	9.7±1.8
Plasma protein binding (bound drug %)	92.4±2.1

C_Lint, *in vitro*_, *in vitro* intrinsic clearance; CL_int, *in vivo*_, *in vivo* intrinsic clearance; MDCK, Madin-Darby canine kidney.

**Table 2:** Pharmacokinetic parameters of LMT-28 after intravenous and oral administration at doses of 5 mg/kg in male BALB/c mice (n = 4)

Parameters	Intravenous (*i.v*)	Oral (*p.o.*)
Dose (mg/kg)	5	5
T_max_ (h)	-	0.80±0.67
C_max_ (ng/mL)	-	137±100
t_1/2_ (h)	1.37±0.29	1.13±0.27
CL (L/h/kg)	8.66±4.51	-
V_ss_ (L/kg)	12.9±4.66	-
AUC_8h_ (h·ng/mL)	661±253	292±202
AUC_∞_ (h·ng/mL)	677±264	302±209
MRT (h)	1.58±0.32	1.9±0.55
F (%)	-	38.2

Data represent mean±standard deviation.

T_max_, the time to reach a peak concentration; C_max_, peak plasma concentration; t_1/2_, terminal elimination half-life; CL, clearance; V_ss_, distribution volume at steady state; AUC_8h_, area under the plasma concentration-time curve values of LMT-28 during 8 h; AUC_∞_, able to cover approximately 96.7% of the total AUC; MRT, mean residence time; F, bioavailability.

## References

[1] Venkatesh S, Lipper RA (2000). Role of the development scientist in compound lead selection and optimization. J Pharm Sci.

[2] Kerns EH, Di L (2003). Pharmaceutical profiling in drug discovery. Drug Discov Today.

[3] Han SY, Lee CO, Ahn SH (2012). Evaluation of a multi-kinase inhibitor KRC-108 as an anti-tumor agent *in vitro* and *in vivo*. Invest New Drugs.

[4] Kerns EH, Di L (2004). Physicochemical profiling: overview of the screens. Drug Discov Today Technol.

[5] Li AP (2001). Screening for human ADME/Tox drug properties in drug discovery. Drug Discov Today.

[6] Scheller J, Garbers C, Rose-John S (2014). Interleukin-6: from basic biology to selective blockade of pro-inflammatory activities. Semin Immunol.

[7] Hunter CA, Jones SA (2015). IL-6 as a keystone cytokine in health and disease. Nat Immunol.

[8] Huang CM, Lee TT (2018). Immunomodulatory effects of phytogenics in chickens and pigs - A review. Asian-Australas J Anim Sci.

[9] Zhao S, Pang Y, Zhao X, Du W, Hao H, Zhu H (2019). Detrimental effects of lipopolysaccharides on maturation of bovine oocytes. Asian-Australas J Anim Sci.

[10] Kumari N, Dwarakanath BS, Das A, Bhatt AN (2016). Role of interleukin-6 in cancer progression and therapeutic resistance. Tumour Biol.

[11] Yao X, Huang J, Zhong H (2014). Targeting interleukin-6 in inflammatory autoimmune diseases and cancers. Pharmacol Ther.

[12] Wolf J, Rose-John S, Garbers C (2014). Interleukin-6 and its receptors: a highly regulated and dynamic system. Cytokine.

[13] Leu CM, Wong FH, Chang C, Huang SF, Hu CP (2003). Interleukin-6 acts as an antiapoptotic factor in human esophageal carcinoma cells through the activation of both STAT3 and mitogen-activated protein kinase pathways. Oncogene.

[14] Jones SA, Scheller J, Rose-John S (2011). Therapeutic strategies for the clinical blockade of IL-6/gp130 signaling. J Clin Invest.

[15] Hong SS, Choi JH, Lee SY (2015). A novel small-molecule inhibitor targeting the IL-6 receptor beta subunit, glycoprotein 130. J Immunol.

[16] Baka E, Comer JEA, Takacs-Novak K (2008). Study of equilibrium solubility measurement by saturation shake-flask method using hydrochlorothiazide as model compound. J Pharm Biomed Anal.

[17] Zhou L, Yang L, Tilton S, Wang J (2007). Development of a high throughput equilibrium solubility assay using miniaturized shake-flask method in early drug discovery. J Pharm Sci.

[18] Park JS, Kim MS, Song JS (2011). Dose-independent pharmacokinetics of a new peroxisome proliferator-activated receptor-gamma agonist, KR-62980, in Sprague-Dawley rats and ICR mice. Arch Pharm Res.

[19] Obach RS (1999). Prediction of human clearance of twenty-nine drugs from hepatic microsomal intrinsic clearance data: An examination of *in vitro* half-life approach and nonspecific binding to microsomes. Drug Metab Dispos.

[20] Davies B, Morris T (1993). Physiological parameters in laboratory animals and humans. Pharm Res.

[21] van de Waterbeemd H, Testa B (2009). Drug bioavailability: estimation of solubility, permeability, absorption and bioavailability.

[22] Lipinski CA (2000). Drug-like properties and the causes of poor solubility and poor permeability. J Pharmacol Toxicol Methods.

[23] Lipinski CA (2004). Lead- and drug-like compounds: the rule-of-five revolution. Drug Discov Today Technol.

[24] Thapa RK, Choi HG, Kim JO, Yong CS (2017). Analysis and optimization of drug solubility to improve pharmacokinetics. J Pharm Invest.

[25] Nelson DR, Zeldin DC, Hoffman SM, Maltais LJ, Wain HM, Nebert DW (2004). Comparison of cytochrome P450 (*CYP*) genes from the mouse and human genomes, including nomenclature recommendations for genes, pseudogenes and alternative-splice variants. Pharmacogenetics.

[26] Azarara M, Afrasibirad A, Farzamikia N, Alijani A, Sakhinia E (2017). The effect of GGCX and CYP4F2 gene polymorphisms in genotype-guided dosing of warfarin in patients with a history of cardiac surgery. J Pharm Invest.

[27] Chen LL, Yao J, Yang JB, Yang J (2005). Predicting MDCK cell permeation coefficients of organic molecules using membrane-interaction QSAR analysis. Acta Pharmacol Sin.

[28] Zahner D, Alber J, Petzinger E (2010). Cloning and heterologous expression of the ovine (*Ovis aries*) P-glycoprotein (Mdr1) in Madin-Darby canine kidney (MDCK) cells. J Vet Pharmacol Ther.

